# Uterine Leiomyoma and Reproductive Tract Infections Detected by Polymerase Chain Reaction

**DOI:** 10.30699/IJP.14.1.33

**Published:** 2018-12-27

**Authors:** Geita Saadatnia, Saadatnia Saremi, Behrouz Salehian, Pirooz Salehian

**Affiliations:** 1 *Dept. of Biotechnology, Iranian Research Organization for Science and Technology (IROST), Tehran, Iran*; 2 *Sarem Cell Research Center, Sarem Women’s Hospital, Tehran, Iran*; 3 *Dept. of Endocrinology, City of Hope National Medical Center, Duarte, California*; 4 *Pathobiology Laboratory, Tehran, Iran*

**Keywords:** Leiomyoma, Chlamydia trachomatis, Cytomegalovirus, Herpes simplex virus, Human papillomavirus, Polymerase chain reaction

## Abstract

**Background and Objective:**

For nearly a century, it has been suspected that reproductive tract infections play an etio- logic role in uterine leiomyoma. However, no epidemiologic study of leiomyoma has used the polymerase chain reaction (PCR) to compare uterine tissues from cases and non-cases, and to investigate associations between uterine leiomyoma and infections detected by PCR.

**Methods:**

In this case-control study, 92 leiomyoma tissues from cases, and 94 myometrial tissue from controls were screened by PCR for cytomegalovirus, *Chlamydia trachomati*s, herpes simplex virus-1, 2, and human papillomavirus typed as 16/18 or another strain. Multivariable analysis used age-adjusted logistic regression, and generalized linear regression as appropriate.

**Results:**

In the uterine tissues of cases and unmatched controls, the prevalence of infection was: cytomegalovirus (32.6%, 7.4%), C. *trachomatis *(23.9%, 37.2%), herpes simplex virus-1,2 (25.0%, 13.8%), human papillomavirus 16/18 (13.0%, 10.5%). Leiomyoma was associated with cytomegalovirus (Odds Ratio (O.R.) 6.10; 95% confidence interval (C.I.), 2.40, 15.55) and *Chlamydia *(O.R. 0.47; 95% C.I. 0.23, 0.97). Likewise, the log count of leiomyoma was higher

with cytomegalovirus (+0.65, 95% C.I. +0.34, +0.95) and lower with *Chlamydia *(-0.71, 95% C.I. -1.12, -0.29).

**Conclusion:**

This first application of PCR to leiomyomata and control uterine tissues from non-cases reveals that cytomegalovirus is associated with the presence, number, and volume of uterine leiomyoma, while *C. trachomatis *is inversely associated with leiomyoma, but only in the absence of cytomegalovirus. Current findings provide preliminary evidence that common reproductive tract infections contribute to the growth and control of at least some cases of uterine leiomyoma.

## Introduction

By menopause, most women will have developed uterine leiomyoma (ta) (UL): according to ultrasound evi- dence, the cumulative incidence of UL by age 50 is nearly 70% among white women and more than 80% among African-American women (1). Asymptomatic in most cases, UL nevertheless causes infertility, pelvic pain, and severe menorrhea in a substantial proportion of women, frequently resulting in surgical treatment (2-4). As a consequence, the economic burden of UL on the healthcare systems is substantial; costing between $5.9 and $34.4 billion annually in the United States alone (5). Despite the high cumulative incidence, significant morbidity, and major burden of UL on health and the economy, the pathogenesis of this disorder is not yet well understood.

For nearly a century, it has been suspected that reproductive tract infections (RTI) play an etiologic role in UL (6). According to a case-control study, the risk of UL increases with the number of self-reported episodes of pelvic inflammatory disease, with discontinuation of the intrauterine device due to infectious complications, and with the frequency of perineal talc use, from which findings it has been hypothesized that local uterine irritants of infectious or noninfectious origin contribute to the development of UL (7). However, it has proven difficult to detect infectious pathogens in UL (8, 9), although one laboratory succeeded in isolating cytomegalovirus (CMV) messenger RNA from the uterine tissue of UL cases (10).

Previous case-control studies of RTI and UL have been limited to self-reported history or serologic evidence of RTI. Specifically, among young African-American women screened for UL using ultrasound, the self-reported history of “ever [having been] diagnosed with Chlamydia” has been associated with reduced risk of multiple UL (11). Among a similar population, serologic evidence of exposure to another RTI, herpes simplex virus (HSV) type 2, has been found to not be associated with the presence, number, or size of UL (12).

Recognizing the need to study whether laboratory-confirmed uterine infections are associated with UL, we screened uterine tissues from pathology-confirmed UL cases and controls by polymerase chain reaction (PCR) for *Chlamydia trachomatis*, CMV, HSV type-1,2, and human papillomavirus (HPV, categorized as 16/18 and non-16/18 strains). These pathogens were selected for study because of their affinity for the reproductive tract, prevalence in the adult female population, potential for persistent and/or asymptomatic infection, and in the case of HPV-16/18, leading causal role in tumors of the uterine cervix and anogenital tract (13).

## Materials and Methods

**Subjects**

Approval to conduct this case-control study was obtained from the hospital’s institutional review board. Eligible for study were patients aged 24-64 years who underwent gynecological surgery at this women’s hospital during the period May 2013-March 2014. The leiomyoma status was determined through preoperative ultrasonography, and UL cases were confirmed by pathology. Written informed consent for the collection of clinical data and uterine tissue (leiomyoma tissue from cases, myometrial tissue from controls) was obtained prior to surgery. Cases included all patients who underwent surgery for pathology-confirmed uterine leiomyoma during the study period, and a similar number of consecutive, unmatched controls without UL who underwent endometrial curettage for abnormal bleeding, unexplained infertility, or uterine cyst. (Sample size is discussed below, under “statistical analysis”.)

**Clinical Data**

For each case, the UL count and the dimensions of the largest UL were abstracted from the pathology report. Where the report indicated “more than 8 UL”, a count of 10 was assigned for the current analysis. The height, width, and depth of the largest UL were converted into a spherical volume. Control subjects were assigned zero values for count and volume of UL. Age at surgery was abstracted from the medical chart. There was no missing data.

**Laboratory Testing**

PCR testing of uterine tissues was performed in mixed batches of cases and controls by laboratory personnel who were unaware of the case-control status of individual specimens. For DNA screening, 25-50 mg sections of formalin-fixed, paraffin-embedded tissue were studied. DNA extraction was performed with the High Pure PCR Template Preparation Kit (Roche, GmbH, Germany), using the Human β-globin gene as a positive control for the extraction. DNA amplification was performed in a PCR thermocycler system (Primus 25 Thermal Cycler, USA) under the following conditions: Fast-Start Taq DNA polymerase activation at 96°C for 3 minutes, followed by 42 cycles of denaturation at 94°C for 60 seconds, annealing at 45°C for 90 seconds, and extension at 72°C for 90 seconds. PCR screening for HSV-1,2, CMV, and *C. trachomatis *was performed using pathogen-specific detection kits (DNA Technology, Moscow, Russia). Each kit included a positive control and distilled water as a negative control. Instead of a kit, initial PCR screening for HPV used consensus primers MY09: 5’CGTC- CMARRGGAWACTGATC3’ (R=A/C W=A/T M=A/T) and MY11: 5’GCMCAGGGWCATAAYAATGG3’

(M=A/C W=A/T Y=C/T) and a 5-µL DNA template added to the master-mix (Cat No: K0171, Fermentas, EU). Specimens that tested positive for HPV were then screened for HPV type 16/18 (DNA Technology, Moscow, Russia).

**Statistical Analysis**

For this unmatched case-control study, sample size was chiefly determined by the availability of cases. Pri- mary risk factors evaluated for univariable and multivariable association with UL were PCR-detected infection with CMV, *C. trachomatis*, HSV-1, 2, HPV type 16/18, and other HPV. Increasing age, being an established risk factor for UL in premenopausal women up to 50 years of age, was taken into account to control potential con- founding of the primary associations. Because women over age 50 were not excluded from the current study, and because age as a continuous risk factor for UL has been documented only up to age 50, the association with years of age was currently analyzed as 2 risk factors: one for women under age 50 and one for women age 50 or older.

Associations with UL status were evaluated using unconditional logistic regression, for which there was 82% power (using 2-sided testing and alpha=0.05) to detect a univariable odds ratio of 2.8 or greater for CMV, and 0.40 or lower for Chlamydia, given the prevalence of these infections among current cases. For risk factors sig- nificantly associated with UL, associations with UL count and volume of largest UL were evaluated using gen- eralized linear regression with Poisson distribution and log link. In multivariable analysis, potential interactions between significant infections were explored and retained where significant (P<0.05). Due to the exploratory nature of the study, p values were not adjusted for the testing of multiple hypotheses.

## Results

Age and RTI prevalence among cases (n=92) and controls (n=94) are summarized in Table 1. Among the cases, the UL count was 1 (n=54), 2 (n=16), 3 (n=5), 4-8 (n=3), or more than 8 (n=14), and the volume of the largest UL was median 92.9 (10th-90th percentile range 4.7-760.3) cubic centimeters.

The extracellular matrix (ECM), composed of proteoglycans (PGs), glycoproteins and collagens, is a well- organized structure with numerous physiological and pathological roles. (3, 4) Versican, a member of the aggre- gating chondroitin sulfate PGs family, is accumulated predominantly in the tumor stroma. Due Subjects without uterine leiomyoma (UL) were assigned zero values for count and volume.

**Table 1:** Characteristics of Cases of Uterine Leiomyoma and Control Subjects

**Variable**	**UL Cases (n=92)** **N (Column %)**	**Controls (n=94)** **N (Column %)**
Age, Years		
24-34	44 (47.8)	60 (63.8)
35-49	46 (50.0)	27 (28.7)
50-64	2 (2.2)	7 (7.5)
Cytomegalovirus (CMV)		
Positive	30 (32.6)	7 (7.4)
Negative	62 (67.4)	87 (92.6)
Herpes Simplex Virus (HSV)-1,2		
Positive	22 (23.9)	35 (37.2)
Negative	70 (76.1)	59 (62.8)
* Chlamydia trachomatis *		
Positive	23 (25.0)	13 (13.8)
Negative	69 (75.0)	81 (86.2)
Human Papillomavirus (HPV)		
Positive for 16/18	12 (13.0)	10 (10.6)
Positive but not for 16/18	8 (8.7)	11 (11.7)
Negative	72 (78.3)	73 (77.7)
Total Pathogens Present		
0	32 (34.8)	43 (45.7)
1	36 (39.1)	30 (31.9)
2	14 (15.2)	17 (18.1)
3	9 (9.8)	4 (4.3)
4	1 (1.1)	0

The likelihood of UL was increased by CMV and decreased by *Chlamydia*; no significant association was de- tected with HSV-1, 2, HPV-16/18, and HPV non-16/18 (Table 2). The interaction between CMV and *Chlamydia *was undetectable in the model of UL likelihood but was significant in the models of UL count and volume of largest UL, indicating that, when both pathogens were present, the association with CMV remained and that it disappeared with *Chlamydia *(Table 3). The likelihood of UL, and the volume of largest the UL increased with age, but only before 50 years of age. (Tables 2 and 3) In contrast, the UL count increased with age to the same extent before and after age 50 (Table 3).

**Table 2:** Associations with Uterine Leiomyoma (N=92 Cases, 94 Controls)

**Risk Factors**	**Univariable** **Odds Ratio (95% CI** * **)**	***P***	**Multivariable** **Odds Ratio (95% CI** * **)**	***P***
CMV				
Positive	6.01 (2.48, 14.57)	<0.0001	6.10 (2.40, 15.55)	0.0001
Negative	1.00		1.00	
*Chlamydia trachomatis*				
Positive	0.53 (0.28, 1.00)	0.05	0.47 (0.23, 0.97)	*0.04*
Negative	1.00		1.00	
HSV-1,2				
Positive	2.08 (0.98, 4.41)	0.06	1.71 (0.75, 3.93)	0.20
Negative	1.00		1.00	
HPV, By Viral Strain				
Positive, Type 16/18	1.22 (0.50, 2.99)	0.67	1.04 (0.38, 2.83)	0.95
Positive, Not Type 16/18	0.74 (0.28, 1.94)	0.54	0.59 (0.19, 1.80)	0.35
Negative	1.00		1.00	
Per Additional Year of Age				
If Aged Under 50 Years	1.08 (1.03, 1.14)	0.004	1.08 (1.02, 1.14)	0.01
If Aged Over 50 Years	0.98 (0.93, 1.04)	0.51	0.86 (0.57, 1.30)	0.47

* CI, confidence interval.

**Table 3:** Multivariable Models of Count and Volume of Uterine Leiomyoma, (N=186*)

	**(log)Count of UL Estimate (95% CI)**	***P***	**(log)cm** **3 ** **Volume of Largest UL, Estimate (95% CI)**	***P***
Mean Value among Women Age 35 and Negative for CMV and *C. trachomatis*	0.55 (0.33, 0.78)	----	4.81 (4.76, 4.85)	----
Difference in Mean Value Associated With: CMV and *C. trachomatis *Status		0.02		<0.0001
CMV-Positive, Chlamydia-Negative (n=24)	+0.65 (+0.34, +0.95)		+0.30 (+0.27, +0.33)	
CMV-Positive, Chlamydia-Positive (n=13)	+0.65 (+0.06, +1.24)		+0.36 (+0.29, +0.44)	
CMV-Negative, Chlamydia-Positive (n=44)	-0.71 (-1.12, -0.29)		-1.38 (-1.44, -1.33)	
CMV-Negative, Chlamydia-Negative (n=105)	0		0	
Per Additional Year of Age				
If Aged Under 50 Years (n=177)	+0.03 (+0.01, +0.06)	0.002	+0.099 (+0.097,+0.101)	<0.0001
If Aged Over 50 Years (n=9)	+0.03 (+0.01, +0.05)	0.002	-0.005 (-0.009, -0.001)	0.01

## Discussion

This is the first case-control study of UL to employ PCR to detect pathogens in uterine tissue. According to this study, the DNA of at least one RTI is present in the uterus of the majority of women undergoing gynecologic surgery, whether or not the procedure is undertaken to treat UL. Moreover, two of the more prevalent uterine infections, CMV and *C. trachomatis*, are significantly and consistently associated with the presence, count, and volume of UL.

The current association of UL with CMV is a novel finding, for which there is supportive biological evidence. In 10 out of 10 UL cases in a pathology study, CMV messenger RNA was isolated from the muscular layers of the uterus, namely from fibroblasts and smooth muscle cells (10). Myofibroblasts, cells of an intermediate phenotype between normal uterine muscle and differentiated fibroblasts (14), are active in both keloid scars and uterine fibroids, disorders of extracellular matrix formation that were recently recognized as having similar ultrastructure and gene expression profiles on microarray (15). In both disorders, myofibroblasts become arrested in the proliferation stage of tissue repair (15). CMV has also been associated with excessive proliferation of smooth muscle cells within blood vessels (specifically, vessels injured during coronary angioplasty, resulting in restenosis) (15). Taken together, these studies suggest that CMV infection of the uterus may promote local myofibroblast activity and tissue proliferation, resulting in one or more UL.

The current inverse association of *C. trachomatis *with UL confirms and expands upon a prior report that mul- tiple UL are less often present among women who report a history of Chlamydia (11). Two other case-control studies of UL did not detect an association with Chlamydia, possibly because subjects in those studies had a very low prevalence of that infection (7, 8). According to our analyses of counts and volume of UL, when both CMV and *Chlamydia *are present, the association with CMV prevails over that with *Chlamydia*. The current study also confirms a recent report that HSV-2 is not associated with UL (12).

We interpret our findings as preliminary evidence that CMV may promote the development of a subset of UL cases and that local response to *Chlamydia *may protect against UL cases not associated with CMV. If this inter- pretation is confirmed by biological studies, then it suggests a novel potential strategy to reduce the incidence and associated morbidity of UL, namely combined vaccination against CMV and *C. trachomatis *early in life. At present, there are no approved vaccines against CMV or Chlamydia. However, Phase II trials have been com- pleted for several vaccines against CMV, one of which is currently in Phase III testing (17). Vaccine develop- ment for Chlamydia is at a much earlier stage, but recent work is promising: mucosal (intrauterine or intranasal) immunization with ultraviolet light-inactivated *C. trachomatis *conjugated to adjuvant nanoparticles has induced long-lived genital protection in both conventional and humanized mice by seeding the uterine mucosa with ef- fector T cells that establish resident memory T cells (18).

A chief limitation of this study is its cross-sectional design, which demonstrates association but does not in- dicate the relative timing of infections and UL. Nevertheless, the case-control design is widely used for the first study of any novel risk factor in order to generate preliminary findings that can inform future prospective studies. Another current limitation is the lack of serologic testing for the same RTI screened in uterine tissue by PCR. A comparison of serologic versus PCR findings would have been useful to identify, for example, subjects previously exposed to C. trachomatis but currently PCR-negative, or those with serologic evidence of CMV but without uterine infection by the virus. In addition, all current cases had UL requiring surgical intervention; thus, it remains to be studied whether current findings are generalizable to UL that are asymptomatic or not suf- ficiently burdensome to require treatment. Case reports have linked some cases of UL to parasites responsible for Chagas’ disease (19) and schistosomiasis (20). For practical reasons, we studied only pathogens commonly found in the reproductive tract of women worldwide. Finally, the current study lacked data on covariates other than age and drew its subjects from a single institution and ethnic group. Confirmatory epidemiological studies without these current limitations are needed.

To establish roles for CMV and *Chlamydia *in UL, confirmatory biological studies will also be required to elu- cidate the mechanisms by which these infections may promote (in the case of CMV) or limit (in the case of *Chla- mydia*) the growth of uterine leiomyoma. It may be informative to investigate potential associations between the CMV and *Chlamydia *status and the recently identified molecular subclasses of UL, which are distinguished by mutually exclusive genetic mutations, including high mobility group AT-hook 2 (HMGA2) rearrangements, mediator complex subunit 12 (MED12) mutations, biallelic inactivation of fumarate hydratase (FH), and type IV alpha 5 and alpha 6 collagen (COL4A5-COL4A6) deletions (21).
